# No NLRP3 Inflammasome Expression in the Ouabain Animal Model of Bipolar Disorder

**DOI:** 10.7759/cureus.24765

**Published:** 2022-05-05

**Authors:** Ali A Farooqui, Yonglin Gao, Megan A Coghlan, Rifaat S El-Mallakh

**Affiliations:** 1 Psychiatry, University of Louisville School of Medicine, Louisville, USA

**Keywords:** brain, nlrp3-inflammasome, ouabain, ouabain model of mania

## Abstract

Introduction

Inflammation is believed to play a role in both bipolar illness and unipolar depression. Markers of inflammation are elevated during acute mood episodes. Specifically, gene expressions of the nucleotide-binding domain and leucine-rich repeat pyrin domain containing 3 (*NLRP3*)-related proteins in peripheral blood have been purported to be upregulated in patients with bipolar disorder. We examined the elaboration of NLRP3 in the ouabain animal model of bipolar disorder.

Methods

The frontal cortex, hippocampus, and basal ganglia tissue from young, male Sprague-Dawley rats who received intracerebroventricular (ICV) ouabain as a model of bipolar disorder or artificial cerebrospinal fluid (aCSF) were examined for NLRP3 utilizing protein immunoblot (Western) analysis.

Results

We could not demonstrate any NLRP3 in rat brain, but NLRP3 was detected in control from mouse brain and lung.

Discussion

This study demonstrates that the manifestation of manic behavior in rats treated with ICV ouabain is not accompanied by elaboration of NLRP3 inflammasome. This raises the question of the primacy of inflammation in the pathophysiology of mania. If these findings are reproduced in this and other animal models of mania, they would raise important questions about whether inflammation is a primary or secondary phenomenon in the brains of subjects with bipolar disorder.

## Introduction

Mood disorders have been linked to immunological changes [1,2]. Specifically, the innate immune system is thought to play a vital role in the pathophysiology of bipolar disorder (BD) as evidenced by elevated levels of proinflammatory cytokines such as tumor necrosis factor alpha (TNF-α), acute-phase proteins, inappropriate microglial activation, and deregulation of the NRF2-KEAP1 system [3,4]. The peripheral and central cytokines have been researched in many studies with promising results [5]. One particular pathway of immune activation involves the nod-like receptor pyrin containing 3 or nucleotide-binding domain, leucine-rich repeat, pyrin domain containing 3 (NLRP3) inflammasome, which cleaves caspase-1 into its active form, and results in the biologically active cytokines IL-1b and IL-18 and in the downstream activation of interleukin (IL)-6 and TNF-α [6-8]. The NLRP3 pathway is seen as important because both IL-6 and TNF-a have been purported to be increased during manic episodes [9]. Post-mortem studies of the frontal cortex of patients with BD have suggested activation of the NLRP3-inflammasome resulting in immune activation [10,11].

Intracerebroventricular (ICV) injections of ouabain in rat is an accepted animal model of bipolar illness with induction of symptoms of mania or depression (although ‘mania’ is usually studied) [12,13]. This is one of the few models that create both mania and depression, which is a requirement for an accurate animal model [14,15]. Additionally, this model is the only model that predicts response to effective medications in this condition [16,17]. As such, the examination of the consequences of sodium pump inhibition would be important to examine. This animal model is associated with significant oxidative stress [18], so it has been surprising that investigations on the inflammatory cytokines in this animal model have not been promising with cerebrospinal fluid, serum, and brain structures not exhibiting changes observed in patients with BD [19]. In this study, we assess the NLRP3 inflammasome expression in the brains of rats in the ouabain animal model of bipolar illness.

## Materials and methods

Animals

Male Sprague-Dawley rats (approximately 200 g) were allowed to acclimate to the animal facility for one week after shipping from the vendor (Charles River Laboratories, Wilmington, USA) prior to the experimental manipulations. All animals were housed individually, on bedding, with free access to food and water and in 12:12::light:dark periods. All behavioral testing was performed during rats' light hours. The project was approved by the Institutional Animal Care and Use Committee of the University of Louisville.

Treatments

Intracerebroventricular cannulae placements into the left lateral cerebral ventricle were performed as previously described [20].** **Briefly, animals received anesthesia with intraperitoneal (IP) ketamine (75 mg/kg) and xylazine (15 mg/kg). Cannulae were placed to a depth of 3.5 mm through a number-60 hole drilled in the dorsal surface of the skull, 2.5 mm lateral and 1 mm caudal to the bregma. Cannulae were fixed in place with dental cement and jeweler's screws and plugged with a wire. Four days after cannulae placement the animals received an ICV injection of 5 µL of ouabain 10−3 M dissolved in artificial cerebrospinal fluid (aCSF), or 5 µl of aCSF alone. The animals were anesthetized and killed by decapitation seven days after ICV ouabain administration. The brain was dissected over ice and the frontal cortex, hippocampus, and basal ganglia were excised and stored separately at −70°C until assay. The current tissue samples had been in storage for approximately 10 years.

Immunoblot analysis

Three distinct tissue sites, from the basal ganglia, hippocampus, and frontal cortex, were obtained from both the ouabain-treated rat brains and aCSF-treated brains. Tissue was homogenized with tissue tearor and sonicated (Heat Systems Microson™ Ultrasonic Cell Disrupter, Farmingdale, USA) over ice in lysis buffer containing 50 mM Tris-HCl (pH 7.4), 1 mM EDTA (ethylenediaminetetraacetic acid), 50 mM NaCl (sodium chloride), 1 mM phenylmethylsulfonyl fluoride (PMSF), 1 % TritonX-100, and 1 % protease inhibitor cocktail (Sigma) for 30 s × 2. The samples were incubated at 4°C for 1 hour and then centrifuged at 13,000 G for 20 minutes. The supernatant was collected. Lowry's assay of protein was run using Bio-Rad DC Kit (BioRad, Hercules, USA) following the manufacturer’s instructions, and absorbance was read at 700 nm. Samples of 100 µg of protein were loaded onto 8% sodium dodecyl-sulfate polyacrylamide gel electrophoresis (SDS-PAGE) gel. After electrophoresis, the gels were electrotransferred onto Immobilon-P transfer membranes (Millipore Corp., Bedford, USA). The membrane was blocked with 5% milk in tris-buffered saline containing 0.1% tween 20 (TBST) at room temperature for 1 hour followed by incubation at 4°C overnight with anti-NLRP3 antibody (1:200, catalogue number ab210491, Abcam, Cambridge, USA) or anti-glyceraldehyde-3-phosphate dehydrogenase (anti-GAPDH)** **antibody (1:20000, Sigma-Aldrich, St. Louis, USA). Lung tissue cell line A549 and short-term frozen mouse brain and lung tissue were used as positive controls. After the membrane was washed with TBST for 15 minutes twice, it was placed with secondary antibody at room temperature for 1 hour and developed with enhanced chemiluminescence (ECL) reagent (Amersham Biosciences Corp., Piscataway, USA).

## Results

**Figure 1:**
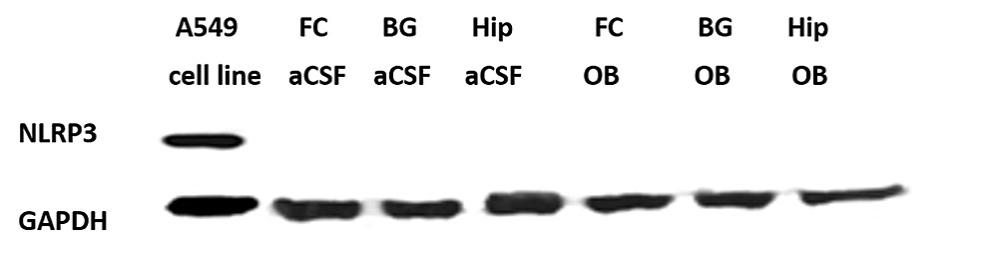
The house-keeping protein glyceraldehyde-3-phosphate dehydrogenase (GAPDH) was present in all samples. Positive control A549 cell line showed expression of NLRP3 at 110 kD when run with samples from the frontal cortex (FC), basal ganglia (BG), and hippocampus (Hip). None of the three control rats (aCSF) or the three ouabain-treated rats (OB) showed expression of NLRP3.

**Figure 2:**
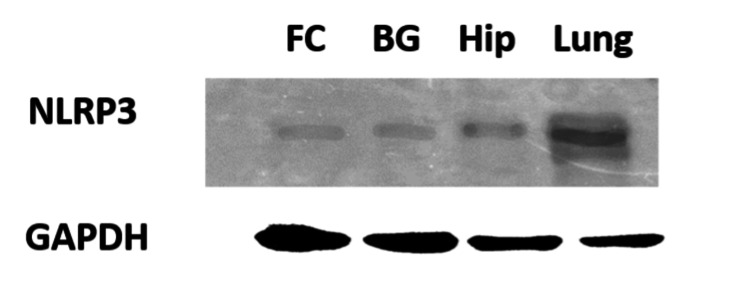
The NLRP3 expression is much lower in the brain tissue of short-time frozen mouse samples compared to lung tissue. FC: frontal cortex;  BG: basal ganglia;  Hip: hippocampus; NLRP3: nod-like receptor pyrin containing 3 or nucleotide-binding domain, leucine-rich repeat, pyrin domain containing 3; GAPDH: glyceraldehyde-3-phosphate dehydrogenase

After two hours of exposure, NLRP3 could not be detected in any tissue from the frontal cortex, hippocampus, or basal ganglia of the rat brain after either aCSF or ouabain treatment (Figure 1). The positive control cell line (A549) did express a clear band at 100 kD (Figure 1). Frozen mouse brain and lung tissue that had not undergone any treatment or surgery and was in storage at -70°C for less than two months had very little NLRP3 (Figure 2). 

## Discussion

A growing body of evidence supports immunological changes associated with BD in human subjects both in the central nervous system and peripherally [1,2]. If these changes can also be seen in animals, it would be easier and faster to study. Animal models of bipolar disorder that alter ion regulation are currently the only models that satisfy all validation criteria for psychiatric animal models [13,15]. Intracerebroventricular injection of ouabain mimics both manic and depressive symptoms in rats and is a well-established and validated model of the illness [15,20]. Yet this model has not shown evidence of significant inflammatory activation [13]. The current study is the first to examine the role of NLRP3 in the animal model of bipolar disorder.

Utilizing antibody protein measurement, we were unable to detect any NLRP3 in either control or experimental rat brain (Figure 1). The absence of NLRP3 in aCSF-treated animals is surprising since other researchers can detect NLRP3 in normal rodent brains [21]. Our positive control utilizing lung tissue cell line, easily detected NLRP3 (Figure 1), and short-term frozen mouse brain control revealed very low levels of NLRP3 versus lung tissue (Figure 2). These data suggest the NLRP3 expression in the brain is much lower than in the lung. Additionally, NLRP3 may not be stable at -70°C for a long period of time. Given the somewhat low levels in the central nervous system, significant elevation of NLRP3 must occur for it to be seen. 

Alternatively, these results might suggest the NLRP3 expression is very low in normal brain and does not become highly activated with ouabain administration in this animal model of mania. This would be unexpected since the NLRP3 pathways are responsive to oxidative stress and the generation of reactive oxygen species (ROS) [22]. Generation of ROS clearly occurs in the ouabain-animal model of bipolar disorder [18]. Furthermore, NLRP3 is responsive to increased intracellular calcium and sodium and reduced intracellular potassium, all of which occur with ICV ouabain treatment [23]. In other words, both NLRP3 activation and cytokine production would be expected in these ouabain treated animals. 

Riegel’s study found oxidative stress markers that occurred in different areas of the brain are varied by ouabain dose and time of administration (immediately after ICV injection and seven days following the ICV injection of ouabain at 10−3 M and 10−2 M) [18]. The current study and Tonin’s study only at day 7 after 10−3 M ouabain ICV administration [19]. It is likely that the dose of ouabain and the time point for the generation of NLRP3 or cytokines are critical. These issues can be corrected in future experiments by detecting different time points after ICV ouabain injection and examining the expression of NLRP3 in fresh brain tissue or in short-term frozen tissue. 

There are limitations in the current study. Of interest, the manufacturer has recently discontinued production of the antibody that we used due to quality control concerns, suggesting other potential issues that could have impacted the results (https://www.abcam.com/nlrp3-antibody-epr20425-ab210491.html). Additionally, despite observations that the ouabain model of bipolar illness is associated with oxidative stress [18], investigations on the inflammatory cytokines have been negative, suggesting that inflammatory biomarkers seen in people are not readily replicated with ICV injections of ouabain in the bipolar animal model [19].

Despite obvious limitations of our study, we believe our findings in combination with those in the literature suggest that this study represents a pertinent negative in the body of literature on the ouabain animal model of bipolar disorder and our understanding of both manic and depressive symptoms. Specifically, while NLRP3 and other inflammatory markers can be seen in mania [24], they do not appear to be necessary for its manifestation in this animal model. Caution is required in the interpretation of data in patients finding proinflammatory changes, and in the interpretation of data from animal models. Specifically, it is important to investigate whether elaboration of inflammatory markers in bipolar disorder is a primary or secondary phenomenon [25]. 

## Conclusions

There is a growing body of literature on the expression of NLRP3 in central nervous system diseases. NLRP3-inflammasome complex and consequent cytokine activation are not seen immediately in the widely utilized ouabain model for mania. Additional research with the ouabain animal model of mania is necessary to clarify these findings. Our results suggest that future experiments of inflammatory markers must examine their presence at different time points after ICV ouabain administration and do so with fresh or short-time frozen brain tissue. If our findings are confirmed, they would raise a question as to the primacy of immunologic activation in human bipolar illness.
